# Knowledge over Time of Action Codes for Perceived Objects: An Exploratory Study on Developmental Children

**DOI:** 10.3390/brainsci14090854

**Published:** 2024-08-24

**Authors:** Marinella Coco, Federica Di Pasquale, Antonello Pellicano

**Affiliations:** 1 Department of Educational Sciences, University of Catania, 95100 Catania, Italy; federicadp1994@gmail.com; 2 B.R.I.T. (Bio-Nanotech Research and Innovation Tower) Service Center, University of Catania, 95100 Catania, Italy

**Keywords:** objects knowledge, action codes, object-based correspondence effect, learning

## Abstract

Over the past 20 years, there has been a growing interest in the processing of tool objects, and in the spatial S-R correspondence effects obtained with pictures of manipulable objects. Beyond the original affordance activation account, a location coding account has been proposed for such behavioral effects, which states that the location of the visually salient portion of an object speeds up spatially aligned motor responses and slows down misaligned ones. Furthermore, an additional action coding account has been proposed, according to which it is the direction of the action of the perceived object (e.g., “pouring tea leftward” when the spout of a teapot is leftward-oriented) that biases motor performance. We investigated this action coding account of S-R correspondence effects by comparing the reaction time (RT) performance of two groups: younger (6 to 9 years old) and older (10 to 13 years old) children. We assumed that knowledge of tool objects and the activation of action codes from object perception is proportional to age. Therefore, a larger correspondence effect was hypothesized for the older relative to the younger children’s group. Consistently, a 34 ms correspondence effect was observed in the older children relative to the younger ones (18 ms). The results support the view that action direction is a constitutive feature of tool objects, which is subject to experience, and thus, to increasing knowledge over time.

## 1. Introduction

Objects in the surrounding world frequently offer salient cues through which humans efficiently coordinate their behavior. Such information is overtly selected and learned to the extent that it enables to respond adaptively to several situations; it is also covertly processed ([1,2]; see also [3]). There is well-established evidence that covert, automatic motor responses are triggered either by the locations of objects [4], or by single components within their structure [5]. Most of the objects in our surroundings are artifacts: tools created to subserve particular purposes through specific designs. In general, tools have two components: a goal-directed portion on one side (e.g., the spout of a teapot, the head of a hammer, the tip of a screwdriver), which makes them readily recognizable or allows one to guess their function; and a graspable portion (a handle), which most frequently protrudes from the opposite side. Evidence of automatic motor activations have been offered through the so-called handle-to-hand correspondence effect. This effect was first demonstrated by Tucker and Ellis, whose original study [6] inaugurated a very productive line of research that, so far, has acquired more than one thousand citations. In the prototypical study, the experimenter instructed the participants to discriminate whether pictures of common objects (e.g., beer mugs, tea pots) were oriented upright or upside-down by performing either a left (e.g., for “upright”) or a right (for “upside-down”) button-press response. These objects had clearly identifiable and protruding handles, so that they were depicted with their handle oriented to the left or to the right side. Although such a component was irrelevant to the task, the participants’ performances were faster and less error-prone when the orientation of the handle corresponded to the location of the responding hand (i.e., corresponding trials) relative to when the handles and responding hands were on opposite sides (non-corresponding trials). Tucker and Ellis interpreted the observed effect as evidence that perceived objects afforded their proper grasp, thus facilitating the performance of the corresponding hand. According to such *affordance activation* hypothesis (see also [7]), the perception of a tool object, beyond recognizing its perceptual features, triggers appropriate manipulative actions consistent with its identity and canonical usage [8,9,10]. These afforded actions involve either the left or right hand depending on the object’s handle orientation (variable affordances) [11,12]. Such object-handling behavior is assumed to be automatically triggered regardless of the observer’s intentions and extends its bias to motor responses different from grasping (e.g., button presses).

Beyond the affordance activation account, a more parsimonious *location coding* account has gained increasing support. Such an account states that the protruding portion of an object is visually salient to the observer and gives the object a spatial orientation. Therefore, it is not the location of the handle itself that drives S-R correspondence, but its salience, which is responsible for visual asymmetry. As such, the jutting portion is coded within a spatial stimulus set (e.g., left and right spatial codes produced by the horizontal orientation of the handle) that overlaps [13] with the spatial response set (left and right task responses) [5,14,15]. In essence, the handle-to-hand correspondence effect consists of a variant of the *Simon effect* [10,16,17,18] named the object-based Simon effect [14] or the object-based correspondence effect [19]; that is, to earlier evidence that task-irrelevant location or orientation of stimuli can affect motor responses. Recently, Azaad, Laham, and Shields [18] performed a meta-analysis of object-based correspondence effects to estimate the overall size of the effect and to test for moderation ascribable to either affordance activation or location coding accounts. They reported a small but significant correspondence effect consistent with the location coding account. Moderator analyses revealed larger effect sizes when the stimuli were silhouettes as opposed to detailed photographs, when they were centered according to their base or graphic distribution on the screen, and when their task-relevant features were not related to their function. Furthermore, the correspondence effects were not moderated by response alternatives administered within or between the hands nor by their interaction with the silhouette, drawing, or photograph versions of the stimuli. In conclusion, since almost fifteen years ago, a large amount of evidence has been produced, consistent with a location coding and against an affordance activation account of object-based correspondence effects.

**Figure 1:**
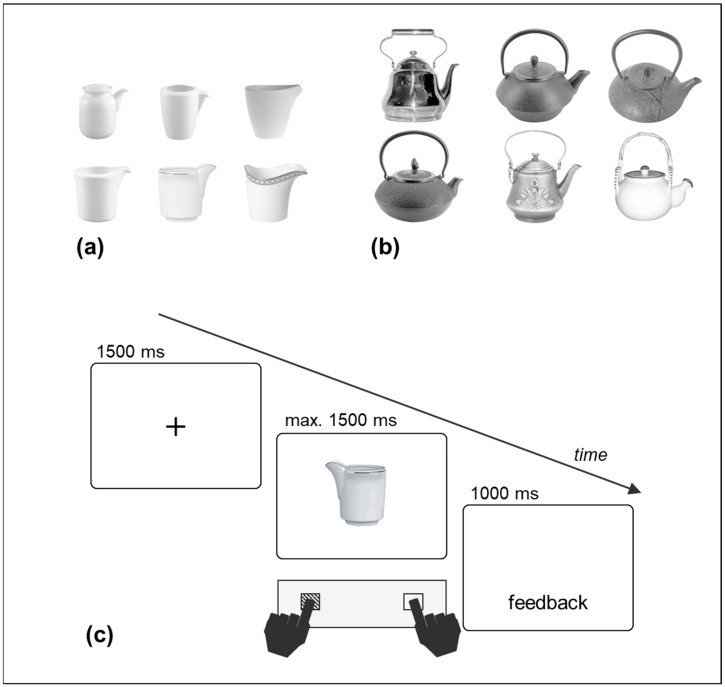
Creamer stimuli (**a**), teapot stimuli (**b**), and the trial structure of the experiment (**c**). The sequence in (**c**) represents a spatial correspondence condition between the orientation of the salient portion of the upright stimulus (the spout) and the location of the button-press response. Original figures in (**a**,**b**) previously used in [20]. Figure in (**c**) modified from [22].

Among these studies, Pellicano and coworkers have devoted themselves to unambiguously address the correspondence effect to location coding or motor affordances by taking advantage of tool stimuli with specific structures. Pellicano, Koch, and Binkofski [20] conceived of two kinds of objects (i.e., creamers and teapots) to separately investigate the effects of their graspable and visually salient portions. For their creamer stimulus set (Figure 1a), the goal-directed portion (the spout) was left–rightward jutting, whereas on the opposite side, the graspable portion was not jutting but integrated into the body of the object. For their teapot stimulus set (Figure 1b), the spout was also the only left–rightward jutting portion, and a handle was jutting from the central–upper side, thus providing no lateral asymmetry. The results of their seven experiments consistently displayed a correspondence effect between the lateral button presses and the location of the spout. Thus, when the objects were asymmetric because of a distinctive portion alternative to the handle, the correspondence effects were driven by this “other” portion, thus clearly supporting the location coding account [20,21]. More interestingly, beyond location coding, Pellicano et al. [20] proposed an *action coding* account of correspondence effects, according to which the spatial codes of tool objects arise from a higher-level process of semantic and action features. Indeed, with the visual processing of a tool, rather than a basic coding of its structural asymmetries, the direction of its proper action is coded (e.g., a leftward pouring action for creamers and teapots with their spout on the left; see Pellicano et al. [20], Experiment 4). Such coding of action direction took place when pairs of objects were displayed (e.g., a teapot and a cup), as well as when objects were displayed alone.

In the present study, we aimed to further explore the action coding account for object-based correspondence effects. We moved from the assumption that action codes plausibly depend on the amount of procedural and semantic knowledge that one is expected to develop on tools across repeated experience over time. Therefore, the capability to code action directions when such tools are perceived, as well as their strength, should be proportional to age. This is most clear for younger populations who are building up their knowledge from zero. Along this line, larger differences in performance should be observed between older children, who have had higher chances of coming across objects and developing longer experience and stronger knowledge, and younger children, who have been able to accumulate less experience. To this purpose, we compared one group of younger children (6 to 9 years old) and one group of older children (10 to 13 years old). If the coding of action direction is part of tool object processing and depends on the experience of the younger participants, then stronger action codes should be developed, and a larger correspondence effect should be observed in the older children relative to the younger children.

## 2. Materials and Methods

### 2.1. Participants

Fifty-two children of elementary and middle school age (6–13 years old) from a summer camp participated in the study. Thirteen of them voluntarily interrupted their participation after the first half of the experiment, and three of them could not complete their task because of technical problems with the hardware. As a result, thirty-six children (21 females and 15 males; mean age of 9.28 years; SD of 2.42 years) entered the study design. All of them were right-handed, measuring +67/100 according to the Edinburgh Inventory of Handedness [23]. All had normal or corrected-to-normal vision, normal color vision, and were naïve as to the purpose of the experiment. The participants were divided into one group of eighteen children younger than 10 years old (+58/100; 10 females with a mean age of 6.70 years and SD of 1.16 years; 8 males with a mean age of 7.75 and SD of 1.28), and one group of eighteen children aged 10 years or older (+75/100; 11 females with a mean age of 11.45 and SD of 1.13; 7 males with a mean age of 11.29 SD of 0.76). The experiment received approval from the local ethics committee (protocol n. 538, 11 January 2024) and was carried out in conformity with the Convention of Helsinki.

### 2.2. Materials

The participants sat in front of a laptop computer at a viewing distance of approximately 57 cm from its 13 inc. color monitor (at such an eye distance, 1 cm on the screen corresponds to 1 degree of visual angle). A standard computer keyboard was connected to the laptop to collect the button press responses. The stimulus presentation, response collection, and timing were controlled by E-Prime Professional v2.0 software (http://www.pstnet.com).

A black fixation cross (visual angle of 0.4 × 0.4 degrees) and the target stimulus were displayed in the center of the screen on a white background. Pictures of six different creamers (Figure 1a), and six different teapots (Figure 1b) were used as the target stimuli.

The structure of the creamers was characterized by a jutting spout on one side and a “flat”, non-jutting graspable portion on the opposite side. The teapots also had a lateral jutting spout and a symmetrical, horizontal handle on the top. The creamers had a visual angle of 4° to 5° in width and 4.2° to 4.8° in height; the teapots ranged from 10.8° to 13.2° in width and 11.5° to 12.2° in height. The stimuli were centered based on their main body, ensuring that the only part extending left- or rightward was their spout.

Each stimulus was depicted in two vertical orientations (upright and upside-down) and two horizontal orientations (spout on the left and on the right side), orthogonally combined with each other, for a total of 24 creamer-and-teapot stimulus combinations.

### 2.3. Procedure

The participants were tested individually in a quiet and softly lit facility of the summer camp. At the start of each trial, a fixation cross was displayed for 1500 ms, followed by the target stimulus, which stayed on the screen for up to 1500 ms or until a response was made. The participants responded as quickly and accurately as possible to the upright or upside-down orientation of the object stimulus, while ignoring its horizontal orientation. The responses were left or right button presses (the “A” and “L” keys of a standard keyboard, marked with colored tape) with the left and right index fingers, respectively. The keyboard was positioned to allow the keys to be equidistant from the body midline. The experimenter read the instructions aloud and ensured that they were understood by the children. Half the children responded to the upright stimuli with the left button and to the upside-down stimuli with the right button, whereas the other half had the opposite assignments. The creamer and teapot stimuli were administered in two separate blocks of trials. Half the children performed the creamer block first, while the other half performed the teapot block first. For correct responses, a blank screen was displayed for 1000 ms. In case an incorrect or a missing response occurred, the messages “ERRORE” (wrong response) or “NON HAI RISPOSTO” (missing response) were shown for 1000 ms (Figure 1c). The participants took short breaks between the trial blocks. The entire experiment lasted approximately 30 min.

### 2.4. Design

The responses were classified as corresponding when the position of the responding hand matched with the orientation of the visually salient part of the object (i.e., its spout), and as non-corresponding when the responding hand was on the side opposite to the spout. The mean correct RTs and arcsin-transformed error rates (ERs) were passed through two repeated-measures analyses of variance (ANOVA), with the *age group* (younger than 10 years old vs. equal to/older than 10 years old) as the between-subjects variable, and *object* (creamer vs. teapot) and *correspondence* (salient portion-to-response position corresponding vs. non-corresponding pairings) as the within-subject variables. All statistical analyses were conducted using SPSS v29 (IBM, Armonk, NY, USA).

## 3. Results

### 3.1. RTs

**Figure 2:**
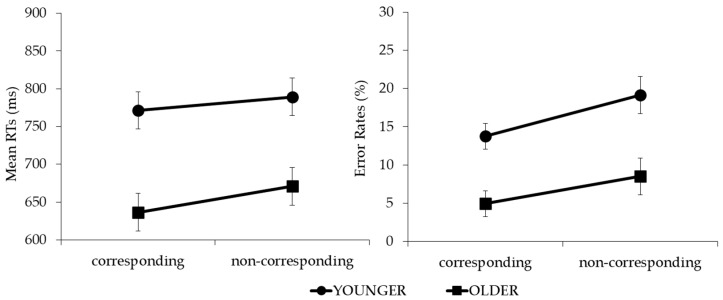
Graphs of age group × correspondence interaction for reaction times (RTs) and error rates (ERs).

Omitted responses (mean of 5.1%) and RTs two SD below (0.7%) or above (6.1%) the individual means were filtered out and not considered for the analyses. The main effect of the age group was significant (*F*(1, 34) = 13.175, *p* < 0.001, η^2^_p_ = 0.28). The younger participants performed slower than the older ones (780 vs. 654 ms). The main effect of the object was not significant (*F*(1, 34) = 0.587, *p* = 0.449, η^2^_p_ = 0.02), displaying no difference between the creamers (724 ms) and teapots (710 ms). The main effect of correspondence was significant (*F*(1, 34) = 46.760, *p* < 0.001, η^2^_p_ = 0.5). The RTs were shorter in the corresponding condition relative to the non-corresponding condition (704 vs. 730 ms). Crucially, correspondence significantly interacted with the age group (*F*(1, 34) = 4.642, *p* = 0.038, η^2^_p_ = 0.12). The salient side-to-response position correspondence effect was significant in both the younger (*t*(17) = 3.060, *p* = 0.007, *dz* = 24.70) and older groups (*t*(17) = 6.987, *p* < 0.001, *dz* = 20.77 (corrected alpha level = 0.025)), and was larger in the older (34 ms) relative to the younger group (18 ms) (*t*(34) = 2.155, *p* = 0.038, *dz* = 22.82) (Figure 2, left panel). No further interactions reached significance (*F*s(1, 34) < 1).

### 3.2. ERs

The main effects of the age group and correspondence were significant (*F*(1, 34) = 15.667, *p* > 0.001, η^2^_p_ = 0.32, and *F*(1, 34) = 7.210, *p* = 0.011, η^2^_p_ = 0.18, respectively) but did not interact with each other (*F*(1, 34) = 0.351, *p* = 0.557, η^2^_p_ = 0.01) (Figure 2, right panel). Overall, the younger group committed more errors than the older one (16.4% vs. 6.7%), and the percentage of errors was lower in the corresponding trials relative to the non-corresponding trials (9.3% vs. 13.8%) (see Table 1 for complete reports).

## 4. Discussion

Over the past 20 years, there has been a growing interest in spatial S-R correspondence effects obtained with pictures of manipulable objects. Beyond the original evidence of S-R correspondence driven by the orientation of handle parts and explained in terms of the activation of grasping affordances [8], more recent evidence suggests that it is rather the orientation of the protruding salient portion of the tool object (and, therefore, the coding of its mere location) that drives the correspondence effect, irrespective of being the handle or not—which is called the object-based correspondence effect [14,21]. More recently, Pellicano, Koch, and Binkofski [20] suggested an action coding account for such a correspondence effect that depends on cognitive, iconic representations of action directions (e.g., the “pouring action of a teapot into a cup”), and not on the lower-level coding of merely protruding parts of visual configurations. This action coding interpretation was applied to both single objects and pairs of objects (see also [22]).

In the present study, we aimed to further investigate the nature of the spatial codes generated from the perception of objects. The same pictures of object stimuli that Pellicano et al. [20] used in Experiment 1A (i.e., creamers) and Experiment 2A (i.e., teapots) and the same behavioral tasks were implemented. Two groups of younger (6 to 9 years old) and older (10 to 13 years old) children were selected, with the assumption that knowledge of tool objects (including those employed in the study) is proportional to age. As a consequence, the older children had higher chances of having come across them and developing more experience, as well as stronger knowledge, than the younger children. This also concerns the coding of the proper action direction for the perceived tool object. We hypothesized that if the higher-level coding of action direction is responsible for the S-R correspondence effect, then a larger effect should be observed in the older children relative to the younger children group.

The results were consistent with our hypothesis: a larger correspondence effect (34 ms) was observed in the older children relative to the younger ones (18 ms). The results support the view first proposed by Pellicano et al. [20], that action direction is a constitutive feature of tool objects, suggesting that it is subject to experience, and thus, to increasing knowledge over time.

As widely observed in the literature, spatial correspondence effects can be obtained with several spatial codes, for example location codes, iconic–symbolic codes (centrally presented arrows pointing left/rightward), or semantic codes (spatial words) [24,25]. More recently, object-based correspondence effects have suggested that, ideally, spatial codes can be formed by any stimulus configuration that provides visual asymmetries [26]. In all these cases, the correspondence effect is attributed to the automatic tendency to respond toward the (salient) source of stimulation [4] when dimensional overlap is provided between the stimulus and response sets [13], and irrespective of whether this response is the correct or wrong one. Such automatic responses can only be partially inhibited in a voluntary way, through cognitive control [16], which can mitigate, to some extent, the interference effect, but cannot neutralize it. Thus, cognitive control mechanisms are well consolidated in adults: they have a role in determining the amount of interference at the response selection stage and ultimately in determining the size of the behavioral effect. Of interest for the purpose of the present study, the investigations of developmental children showed that the size of the interference effects decreased from age 6–7 to 8 years and reached adult-like levels within the 10th year of life [27,28,29,30]. This suggests that cognitive control develops rapidly from 6–8 years of age and is established at around the age of 10.

Crucially, our results went in the opposite direction: the correspondence effect was smaller within the age range in which cognitive control is expected to be weaker (and the effect was larger) and larger in the older age range, when cognitive control should be well established. This is consistent with the view that, action codes were formed instead of basic location codes. Differently from the latter, the strength of the action codes (on top of the same cognitive control mechanisms) was proportional to increased knowledge across time.

## 5. Conclusions

In conclusion, we have provided additional support to the view that action direction codes can be formed for the perception of objects which are independent of an inner motor simulation within the observer’s motor system (functional affordances). As such, our results suggest that the strength of these codes increases with increasing long-term semantic, episodic, and/or procedural knowledge of common use tool objects.

## 6. Limitations

We experienced many children dropping out due to fatigue or loss of interest in the task. The resulting sample size was, therefore, smaller than the available pool of participants. Despite statistical power reduction, we still obtained significant effects that were, in our opinion, informative of underlying cognitive processes. In the follow-up investigations, we prospect to increase the sample size.

## Figures and Tables

**Table 1:** Mean RTs and ERs for S-R corresponding (C) and non-corresponding (NC) conditions in younger and older children.

Children	RTs (ms)			ERs (%)		
	C	NC	Effect Size	C	NC	Effect Size
Younger (6–9 years old)	771	789	18	13.7	19.1	5.4
Older (10–13 years old)	637	671	34	4.9	8.5	3.5

## Data Availability

The original contributions presented in the study are included in the article. Further inquiries can be directed to the corresponding author.
